# Music, Language, and The N400: ERP Interference Patterns Across Cognitive Domains

**DOI:** 10.1038/s41598-020-66732-0

**Published:** 2020-07-08

**Authors:** Nicole Calma-Roddin, John E. Drury

**Affiliations:** 1 Department of Behavioral Sciences, New York Institute of Technology, Old Westbury, New York, USA; 2 School of Linguistic Sciences and Arts, Jiangsu Normal University, Xuzhou, China; 3 Present Address: Department of Psychology, Stony Brook University, New York, USA

**Keywords:** Cortex, Attention, Language, Long-term memory

## Abstract

Studies of the relationship of language and music have suggested these two systems may share processing resources involved in the computation/maintenance of abstract hierarchical structure (syntax). One type of evidence comes from ERP interference studies involving concurrent language/music processing showing interaction effects when both processing streams are simultaneously perturbed by violations (e.g., syntactically incorrect words paired with incongruent completion of a chord progression). Here, we employ this interference methodology to target the mechanisms supporting long term memory (LTM) access/retrieval in language and music. We used melody stimuli from previous work showing out-of-key or unexpected notes may elicit a musical analogue of language N400 effects, but only for familiar melodies, and not for unfamiliar ones. Target notes in these melodies were time-locked to visually presented target words in sentence contexts manipulating lexical/conceptual semantic congruity. Our study succeeded in eliciting expected N400 responses from each cognitive domain independently. Among several new findings we argue to be of interest, these data demonstrate that: (i) language N400 effects are delayed in onset by concurrent music processing only when melodies are familiar, and (ii) double violations with familiar melodies (but not with unfamiliar ones) yield a sub-additive N400 response. In addition: (iii) early negativities (RAN effects), which previous work has connected to musical syntax, along with the music N400, were together delayed in onset for familiar melodies relative to the timing of these effects reported in the previous music-only study using these same stimuli, and (iv) double violation cases involving unfamiliar/novel melodies *also* delayed the RAN effect onset. These patterns constitute the first demonstration of N400 interference effects across these domains and together contribute previously undocumented types of interactions to the available pool of findings relevant to understanding whether language and music may rely on shared underlying mechanisms.

## Introduction

Over the past decades a fruitful body of research examining language and music processing has shown we have at least as much to learn from joint investigations of their relationship as we do from studying either in isolation^1–5^. One such line of investigation concerns the extent to which the processing of language and music may be subserved by shared brain mechanisms or whether these systems are rather supported by independent modular systems^4,6–10^. Consider in this connection the notion that linguistic and musical cognition may be supported by shared syntactic processing resources^1,8,9,11,12^. Accounts of these domains as cognitive systems each require reference to sequentially and hierarchically organized internal representations (i.e., *a syntax*^13–16^). Proponents of shared syntactic processing maintain that while language/music involve different constituent vocabulary (i.e., words, morphemes vs. notes, chords, etc.), complex structured representations in these domains may be supported by some shared or overlapping set of processing resources.

In the present study we pose the question of whether other components of language and music processing may be shared, in particular the mechanisms responsible for managing access/retrieval of information from long term memory (LTM). To this end, we pursued an approach drawing upon previous work probing language and music syntactic processing with interference paradigms^17–19^, in particular experiments that measure event related potentials (ERPs^12,20^). Such studies typically involve simultaneous processing of language and music stimuli, for example, word-by-word sentence reading while listening to chord progressions (or melodies) with the presentation of each chord (or note) time-locked to the presentation of each word. Coupled with electrophysiological measurements, such studies make it possible to observe brain response profiles to manipulations disrupting either the music or the language processing streams independently, or both together (double violations). Interactions that arise in such paradigms have been interpreted as indexing simultaneous demands imposed by the two processing streams upon some common set of underlying neural resources.

Importantly, ERP studies that have investigated syntactic violations within each domain *separately* have demonstrated response profiles that are at least superficially similar. In language, disruption of syntactic processing with violations involving major word category (e.g., encountering a verb in a context where only nouns are possible) or incorrect inflectional/morphological marking (see reviews^21,22^), often elicit a biphasic response consisting of an anterior negative-going deflection (“left anterior negativities” or “LANs”) followed by a relative positivity over posterior regions (P600s). In music, similar biphasic violation responses involving anterior negativities and P600s can be elicited by disruptions of harmonic processing (e.g., out-of-key notes in melodies or incongruent continuations of chord progressions^12,20,23–25^). The musical correspondent of linguistic LAN effects are referred to as “RANs” (for “right anterior negativities”), given that the first report of these effects demonstrated a right lateralized scalp distribution^23^ (note we do not, in the present report, distinguish between earlier (“ERAN”) versus later onset RAN-type effects; our view is that there is no such functional distinction, similar to the analogous effects in the language domain; see Steinhauer & Drury^22^).

Although the mere similarity of linguistic LAN and musical RAN does not make a case for overlap of underlying neural systems, when music and language syntactic violations are encountered together in interference paradigms, linguistic LAN effects have been shown to be attenuated^12^. Furthermore, available data suggest this type of ERP interaction profile is specific to simultaneous *syntactic* disruptions. For example, in Koelsch *et al*.^12^ ERP differences were also compared between low and high probability sentence-final words, a manipulation introduced to elicit a language N400 response linked to expectancy/congruency of incoming words (see Kutas & Federmeier^26^ and below). Interestingly, the language N400 response appears *not* to be influenced by concurrent music syntactic violations^12,20^, (a finding that we replicate here with unfamiliar melodies – see below).

The target of the present investigation is the nature of the relationship between language and music long term memory access/retrieval mechanisms^27–29^ as viewed through the lens of the N400 ERP response^26,30^. The motivation for our study relates to the apparent selective influence of music syntactic violations on linguistic LAN effects, while language N400 responses seem immune to such disturbance^12,20^. Here we pose the question of whether this language/music dissociation would hold in circumstances where N400 effects are elicited by both processing streams simultaneously. Note that while we presuppose a particular view of the N400 as indexing semantic LTM access/retrieval mechanisms, the work reported here is ultimately neutral on this topic and could be reinterpreted under other conceptions of this ERP response (we reiterate this point below, and see our Discussion).

### N400 effects in music processing?

In contrast to linguistic LAN effects, the N400 is a negative-going ERP component with a broad scalp distribution, typically maximal in amplitude over central/parietal electrodes, which peaks around 400 ms post-stimulus onset. In language ERP research this component has been argued to reflect the access/retrieval (or binding) of conceptual semantic information stored in long term memory^26^. Alternatively, the N400 has been discussed as indexing semantic integration^31,32^ or inhibition^33^ (and, as just noted above, should either of these alternative conceptions turn out to be correct, this would change how we interpret the results reported here, but this does not affect the core empirical findings; see Discussion).

Though the initial discovery of the N400 was linked to visual language processing^30^, subsequent research has revealed a wider family of N400-like effects that can be elicited across modalities and can be triggered in connection with a fairly breath-taking variety of different types of stimulus manipulations across cognitive domains^26,34^. This makes the N400 an ideal target for studying a wide array of issues in cognitive processing, but this also exposes a curious gap when we consider music cognition. Though the N400 effect has been leveraged in previous studies to examine associative priming between language and music^33–38^, to our knowledge there is only *one* report documenting an N400-like effect elicited by manipulations of auditorily presented music stimuli *in isolation*^24^ (though see other possible cases^39,40^).

Moreover, even in the lone study reporting a music related N400 response^24^, the interpretation of the observed ERP pattern as a *bona fide* N400 is significantly hedged (for reasons we will canvas below). Before turning to that study, note that while previous work makes it clear that N400s *can* be elicited by music stimuli, it is important that such findings have involved cross-domain associative and affective priming^33–38^. While such findings are significant, as they make it clear that music stimuli can be linked to (arguably abstract, amodal) conceptual representations, it is not obvious how this could be employed to create a *violation paradigm* to examine cross-domain interference between language and music involving the neural mechanisms underlying the N400 (similar to the work on syntax mentioned above).

This leads us to Miranda and Ullman (2007) ^24^, (henceforth: “M&U”), where ERP responses were examined for out-of-key notes in both familiar and matched unfamiliar melodies, and for incorrect but in-key notes in familiar melodies. As the present study makes use of the same music stimuli employed by M&U (see §4), we summarize their findings in detail as we expected (most of) their reported patterns to replicate.

First, M&U found that out-of-key notes in both melody types elicited RANs, P600s, and late anterior negativities (interpreted as so-called N500 effects, claimed to index processing of “intramusical meaning”^41,42^, but see Featherstone *et al*.^43^). Second, only familiar melodies elicited a frontal positivity, interpreted as a P3a effect (indexing attention capture^44^), and the familiar melody P600 violation effect was larger than it was for unfamiliar melodies (replicating earlier findings^39^). Third, and most importantly, a posterior negativity for familiar melody violations *only* was also reported, which (for the out-of-key condition) overlapped in timing with the RAN effect but was slightly later in onset. This posterior negativity also manifested for incorrect but in-key notes in the familiar melodies. The scalp distribution of the posterior negativity, coupled with the fact it was elicited only for unexpected notes (whether in-key or out-of-key) in melodies that must be encoded in long term memory, drove M&U to label this negativity, tentatively, as an N400 (which they append with a ?-mark).

Interpreting their “N400?” as a *bona fide* species of N400 makes sense: in the case of familiar melodies, the processes involved in access/retrieval of long term memory representations could be disturbed by unpredicted incongruent continuations in ways that may be analogous to what happens in language when semantically unexpected or incongruent words are encountered. This could be taken to be consistent with some findings in neuroimaging studies that have jointly investigated semantic memory in language and music, where some have argued for partial overlap of underlying networks across these and other cognitive domains^27–29^.

However, inspection of M&U’s data, in combination with previous findings that failed to elicit music N400 responses^45,46^, leads one to be sympathetic with the hesitancy exhibited in M&U’s interpretation of this response pattern. While their “N400?” is consistent across their in-key and out-of-key familiar melody violations, the effect did not exhibit the classic centro-parietal scalp distribution typical of the N400, but rather manifested a fairly restricted parietal/occipital distribution. This may have be due to the fact that this effect coincided with the large amplitude anterior P3a, perhaps canceling out what would otherwise have been a more prototypical N400 distribution. However, M&U’s follow-up analyses examining musicians/non-musicians separately showed that the musicians were driving the P3a effect, which was attenuated in the non-musicians. If the P3a was cancelling a music N400 effect that would otherwise have exhibited a broader distribution, we might have expected it to show just such a broader pattern in the non-musicians (where the P3a was absent). Instead, both cases of the “N400?” exhibited the more restricted parietal/occipital distribution, suggesting two possibilities: first, less musical expertise may result in *both* the absence of the P3a *and* a smaller, more restricted music N400 effect. A second possibility is that M&U’s “N400?” is simply differently distributed than linguistic N400 effects. After all, N400-like responses in other nonlinguistic domains^26,34,47^ can exhibit scalp distributions different from the canonical cases of the language N400 as well.

In any case, M&U’s study is *unique* in having documented a possible music analogue of the linguistic N400 that may be independent of associative or affective cross-domain priming. However, it is important to note that many of the melodies used by M&U had lyrics associated with them. This raises the question of whether melody familiarity is, on its own, sufficient to elicit their N400? effect, or whether this finding may be restricted to cases where melodies are stored in long term memory together with linguistic information (lyrics). However, whether this is the case or not is an issue that is secondary to the main goals of the present study. The aim of our work is to determine whether the posterior negativity seen for familiar melodies is indeed best understood as belonging in the N400 family, and whether simultaneous elicitation of such responses across language and music will demonstrate interference profiles similar to what has been previously shown for syntax. Showing this to be the case would be significant, opening up a range of possible follow-up work examining further details of such response patterns in ways that could shed some light both on the nature of the N400 and the underlying mechanisms that it is thought to index. Further, documenting interference effects for this component would contribute an important and previously undocumented type of interaction to the inventory of findings relevant to understanding how language and music may rely on shared underlying neurocognitive mechanisms.

### Present study

We reemployed M&U’s familiar/unfamiliar melody stimuli in a language/music interference paradigm. Our goal was to both separately and simultaneously elicit music and language N400 effects in a within-participants experimental design, and examine the question of whether these responses might show interaction profiles analogous to those previously reported for manipulations that trigger linguistic LAN and music RAN effects. More generally, for many (if not most) instances where N400-like effects have been reported across different cognitive domains, it has yet to be shown that the underlying systems involved are the same, as opposed to functionally similar but nonetheless distinct neural systems^47^.

To accomplish this, following previous interference studies of language/music syntax, we constructed sentences manipulating conceptual semantic congruency at the VERB position in relative clauses (e.g., …*the ball that the boy has* KICKED/*BAKED). These contexts for our critical target words were chosen so that we could test these same materials in a second experiment inducing morpho-syntactic violations (e.g., …*the ball that the boy will* *KICKED…). (Note: The present study constitutes the first stage of a larger scale multi-part investigation. The syntactic violations follow-up experiment, and between-group comparisons to single modality effects elicited by the language and music stimuli employed here, as well as an investigation of individual differences and item analyses probing familiarity, will be detailed in a longer forthcoming report.)

These target noun-phrase + relative clause fragments were aligned to musical notes in familiar and unfamiliar melodies, using (a subset of) the stimuli from Miranda and Ullman (2007, see §4). Here we only tested the correct/out-of-key contrasts. Target verbs that constituted either correct or violation continuations were time-locked to the target notes in the melodies, and complete sentences were constructed around these frames so that words appeared with all notes in the melodies. The result of these manipulations was a 2 × 2 design shown in Fig. 2 (center panel), which we crossed with melody familiarity to yield the 2 × 2 × 2 design with eight experimental conditions.

### Predictions

Music violations should yield M&U’s patterns: (i) RAN and P600 effects for both familiar and unfamiliar melodies; (ii) only familiar melody violations should elicit their N400? response. It is important to note here that M&U’s music-only study involved a timbre-change detection task, so their participants were attending to the melodies in a task-relevant way. In the present study, participants were tasked with acceptability judgments of the visual/linguistic sequences only, as in previous interference studies targeting syntax^12^. However, RAN/P600 violations to music have previously been shown in designs like ours where task-relevant attention was directed towards the language stimuli only. Therefore, we can reasonably expect to elicit these responses here, though it is possible that such effects may also be suppressed in situations where music is unattended ^48^; (see also Royle *et al*.^49^ for a general discussion of task effects).

Further, we suspected (but did not strongly predict) that the P3a effect previously observed for familiar melodies may not arise here since directing task-relevant attention *away* from the music stimuli and *towards* the complex visual stimuli (sentences) might suppress this response. If so, this design could then also confer the additional possible benefit of allowing a clearer measurement of the music N400 effect to the extent that its distribution may have been partly canceled by the P3a in M&U’s data, as discussed above.

We did not have a clear expectation for the music N500. Previous work indicates that this effect may not consistently arise^50^ or may be reduced in amplitude^48^ when task demands direct attention away from the musical/auditory stimuli. However, should we succeed in eliciting an N500, given that there is an indication from one prior interference study^50^ that semantic manipulations in language may interact with this response, this was an anticipated possible outcome in the present study as well (though, as we show below, no such interactions obtained).

We also predicted the linguistic violations would elicit reliable N400s, possibly followed by later anterior negativities and/or P600s. These two post-N400 effects have been occasionally reported elsewhere in the literature for conceptual semantic violations^51–53^. However, given that such effects are less widely reported and not well-understood, we did not have any prediction regarding whether they would emerge in the present study (see Steinhauer *et al*.^51^ for discussion).

Finally, the important open question to be addressed here is whether language/music interaction effects would obtain (Correctness x Key interactions). Previous work has suggested that music syntactic violations should not influence language N400s (e.g. Koelsch *et al*.^12^ and Steinbeis & Koelsch^50^), so we expected that at least in our unfamiliar melody conditions that finding would replicate (where only main effects of Correctness and Key are expected, with no interactions in the N400 latency ranges).

However, it is an open question whether (Correctness x Key) interaction effects obtain when N400 effects are elicited in both domains simultaneously, as could be the case for the familiar melody conditions. In fact, previous attempts to carry out experiments much like M&U’s or ours found neither music N400 effects^39,45,46^ nor language/music interactions^54^. Thus another possible outcome here would be patterns consistent with this earlier line of work, which is to be expected if the processing of melody familiarity does not in fact engage any systems in common with those underlying language N400s.

## Results

### Sentence final acceptability & melody familiarity

**Figure 1:**
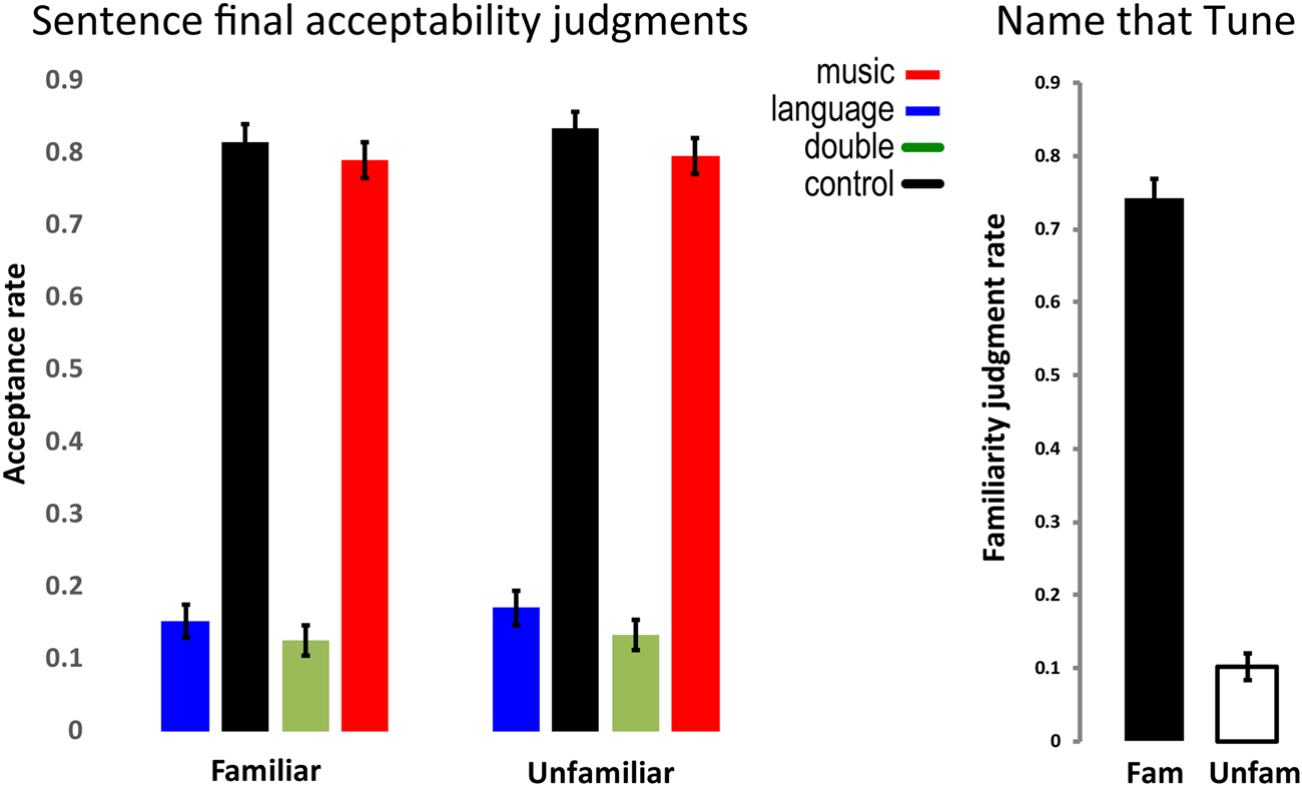
Behavioral responses for end-of-sentence judgment (left) and Name That Tune task (right). Error bars represent 95% CIs.

End of sentence judgment responses (Fig. 1A) yielded main effects of correctness [F(1,35) = 369.45, *p* < 0.0001] and key [F(1,35) = 14.05, *p* = 0.0006], but no effects of familiarity and no interactions. Thus, music violations reduced acceptance rates for both correct sentences (interference) and incorrect sentences (facilitation). *Name That Tune* task responses indicated familiar melodies were, in general, known to our participants (Fig. 1B), yielding highly significant differences between melody types [F(1,35) = 741.91, *p* < 0.0001].

### Event-related brain potentials

**Figure 2:**
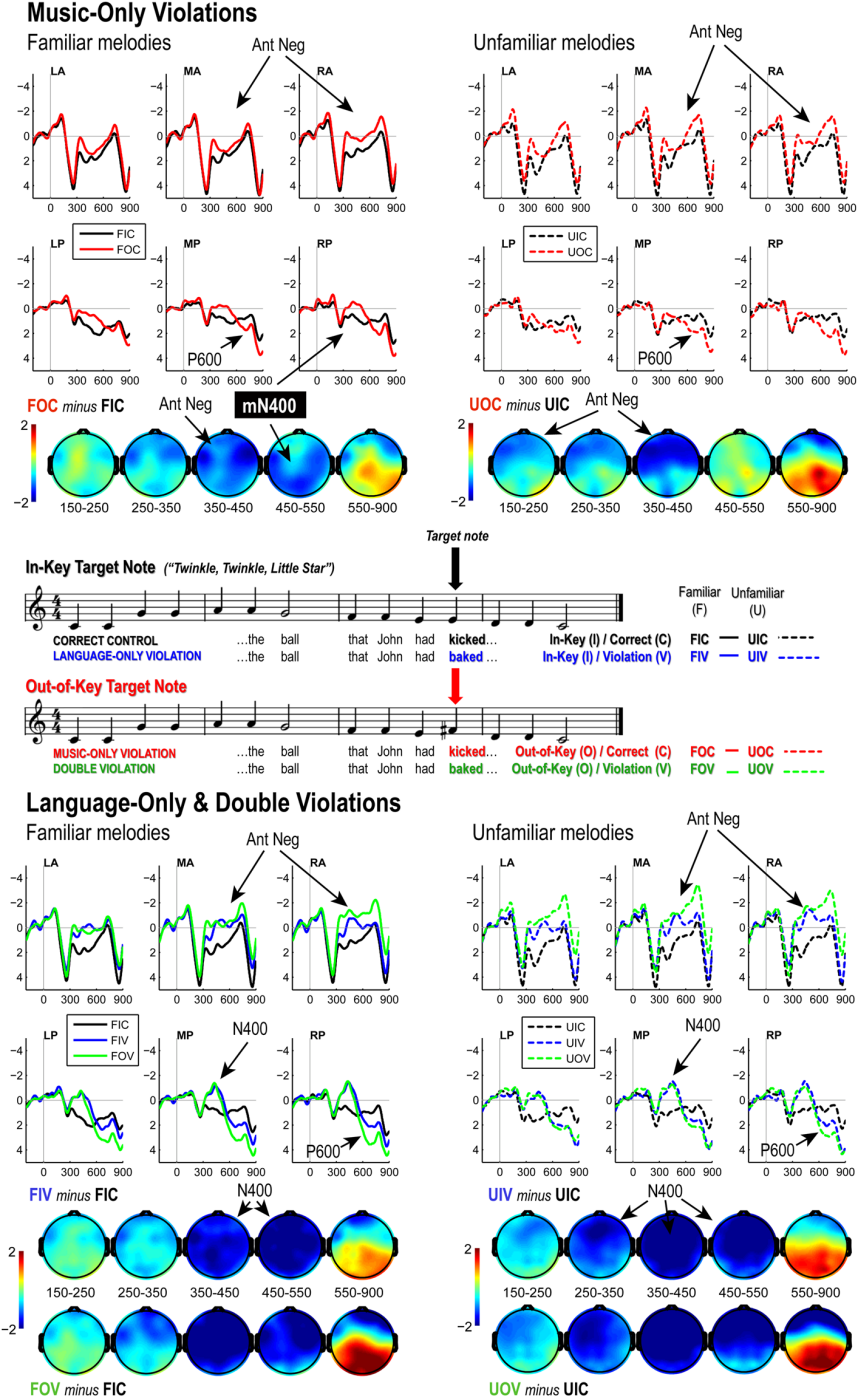
Grand average ERPs (all conditions) and scalp difference maps (Violation minus Control). Music violations shown top panel; Language and double violations in bottom panel. Familiar (lefthand plots) and Unfamiliar (righthand) melody conditions plotted separately. Note that *independent* music and language N400 responses would be expected to yield a larger such effect for the familiar melody double-violation condition (solid green, bottom left) due to additive combination with the music N400. Instead, a sub-additive pattern obtains, indicated by the very similar N400s for language violations whether concurrent music violations obtained (green) or not (blue).

Grand average ERPs in Fig. 2 show all comparisons against matched control items, separating music-only violations (top panels) from language-only and double violations (bottom panels).

Visual inspection of the ERPs in Fig. 2 demonstrates expected early evoked responses (superimposed auditory and visual N1/P2), followed by anterior negativities, prominent N400 responses, and P600 effects for the violations compared to control conditions. Crucially, we succeeded in eliciting N400 effects for the language violations (bottom panels of Fig. 2) as well as the expected N400-like negativity for familiar melody key violations (Fig. 2 top left). In addition, both music and language violations elicited later anterior negativities and P600s.

Observe that the N400 responses in the bottom panels in Fig. 2 are remarkably similar for familiar and unfamiliar melody conditions: both show apparent equivalent N400 responses for language only and double-violation conditions. However, if the apparent music N400 effect for familiar melody music only violations (top left panel) corresponded to independent underlying neural generators, the observed pattern is *not* what we would expect. Instead, what should be seen in that case would be an additive pattern yielding a larger N400 for the familiar double violation case compared to the language only N400 (i.e., the music only and language only N400 responses should sum – *but they do not*). That is, the uniformity of N400 responses in the bottom panels of Fig. 2 clearly reveal a sub-additive N400 response for familiar double violations. This should (and does) yield a significant Correctness x Key interaction in our statistical analysis (see below).

The foregoing patterns were confirmed by a top-down analysis of variance of mean amplitude effects for successive time-windows (following M&U^24^), probing for effects and interactions of Key and Correctness, and interactions of these condition factors with scalp location (see Methods). We present these analyses for familiar and unfamiliar melodies in four parts, showing early RAN effects (150–350 ms), linguistic N400s (350–450 ms), our replication of the music N400 response and the demonstration of the crucial Correctness x Key interactions (450–550 ms), and late posterior positivities and anterior negativities (550–900 ms). Finally, supplementary analyses were conducted to probe language N400 onset latencies (see Methods), which turned out to vary as a function of melody (un)familiarity (see below). Additional analyses including the factor Familiarity are included where relevant, in particular in demonstrating the presence of the music N400 response for familiar melodies, and its absence in unfamiliar ones.

#### 150–250 & 250–350 ms

Familiar melodies showed no significant effects or interactions involving either Key or Correctness in these first two time-windows. There was an indication of the onset of an anterior negativity for familiar melody violations in the 250–350 ms time-window, as can be seen in scalp difference maps (Fig. 2, top left), but the effect of Key was not significant, even when analyses were restricted to anterior regions of interest (ROIs, *Methods*) [F(1,35) = 2.85, *p* = 0.1004].

In contrast to the familiar melodies, within both these two initial time-windows unfamiliar melodies demonstrated Key x Laterality (Lat) interactions [*150–250 ms*, F(2,70) = 5.03, *p* = 0.0200; *250–350 ms*, F(2,70) = 3.78, *p* = 0.0432] and interactions involving Key, Correctness (Cor) and both Anterior/Posterior (AP) and Laterality [*150–250 ms*, Key x Cor x AP x Lat: F(2,70) = 3.31, *p* = 0.0451; *250–350 ms*, Key x Cor x AP: F(1,35) = 5.70, *p* = 0.0225].

Decomposition of these interactions by ROIs showed that when sentences were Correct, unfamiliar melodies elicited a broadly distributed anterior negativity from 150–250 ms yielding a main effect of Key over all anterior ROIs [F(1,35) = 5.29, *p* = 0.0275]. However, while significant effects of Key obtained for the left anterior ROI [F(1,35) = 9.63, *p* = 0.0038], the effect Key was borderline for the mid-anterior [F(1,35) = 4.07, *p* = 0.0514] and right-anterior ROIs [F(1,35) = 2.89, *p* = 0.0978]. Posterior ROIs showed no effects or interactions involving Key when sentences were Correct [F’s <1]. Also, corresponding analyses restricted to sentences with language violations revealed no effects of Key either [F’s < 1].

From 250–350 ms, analyses within the anterior and posterior ROIs separately showed the same general pattern. When sentences were Correct, there was a significant effect of Key over anterior ROIs [F(1,35) = 4.88, *p* = 0.0337] that was absent when sentences contained violations [F < 1]. There were no effects of Key for the posterior ROIs [F’s < 1] regardless of presence/absence of language violations.

In addition, in this same 250–350 ms time-window, language violations paired with unfamiliar melodies yielded a main effect of Correctness [F(1,35) = 13.07, *p* = 0.0009], due the emergence of an N400 effect (Fig. 2, bottom right).

Thus, in these first two time-windows, unfamiliar melody conditions revealed both language and music violation effects, as well as an unanticipated language/music interaction in the form of an apparent suppression of the RAN effect in the double-violation case. During this same period, familiar melody violations showed neither language nor music violation effects.

#### 350–450 ms

Here, robust effects of Key and Correctness were evident for both melody types. The language N400 effect that began in the prior time-window for the unfamiliar melody conditions reached its peak, yielding a main effect of Correctness [F(1,35) = 35.27, *p* < 0.0001]. Similarly, the anterior negativity that was already present for unfamiliar melodies between 150–350 ms became larger, producing a Key x AP interaction [F(1,35) = 6.44, *p* = 0.0158]. Follow-up analysis confirmed the continued anterior distribution of this negativity for unfamiliar music violations as an effect of Key was evident over the anterior ROIs [F(1,35) = 12.15, *p* = 0.0013] but only weakly over posterior ROIs [F(1,35) = 3.46, *p* = 0.0712].

**Figure 3:**
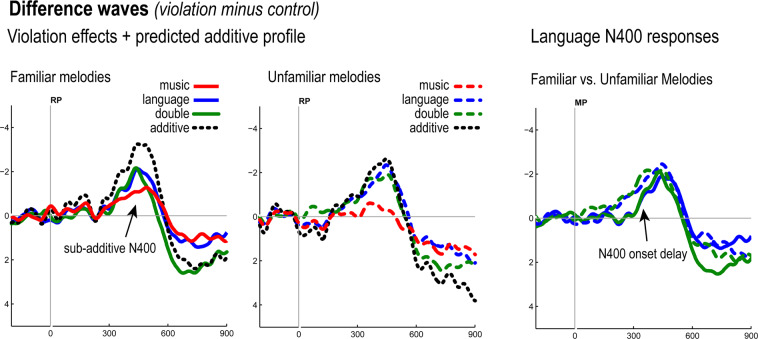
Difference waves for all violations for familiar (left) and unfamiliar (center) melody conditions plotted against predicted additive response profile for double violation conditions; N400 responses for language and double violations superimposed for familiar and unfamiliar melody conditions (right). Note the double-violation (green trace) is sub-additive – corresponding to the Correctness x Key interaction (see also Fig. 2, and main text report of 450–550 ms time-window findings).

Like the unfamiliar melody conditions, familiar melodies also yielded a main effect of Correctness [F(1,35) = 22.49, *p* < 0.0001] as a result of the emergence of the language N400 effect. Given the difference in the onset latency of language N400 effects as a function of melody familiarity (present already in the previous 250–350 ms window for unfamiliar, but not familiar melodies), we examined (50%) fractional peak latency, peak latency, and peak amplitude measures using a jackknife procedure (see *Methods*), comparing N400 difference waves (Violation – Correct) for familiar versus unfamiliar melodies, both with and without concurrent music violations. These analyses revealed no significant differences in peak latency or amplitude for language N400 effects, but fractional peak latency measures revealed a significant main effect of Familiarity [adjusted F(1,35) = 5.68, *p* = 0.0227], with unfamiliar melodies showing an onset ~50 ms earlier on average (compare scalp difference maps Fig. 2, bottom panels, and right hand plot of language violation effect difference waves in Fig. 3). There was no effect of the presence/absence of music violations on this N400 onset delay.

The familiar melody music violation effect in this time window was differently distributed on the scalp – while the Key x AP interaction evident for unfamiliar melodies was not significant for familiar ones [F < 1], there was a Key x Laterality interaction [F(1,35) = 3.45, *p* = 0.0374]. Follow-ups within the levels of Laterality were consistent with a somewhat right lateralized effect of Key, which was weakly present over the left lateral ROIs [F(1,35) = 3.92, *p* = 0.0558] but significant over both the midline [F(1,35) = 4.71, *p* = 0.0369] and right lateral ROIs [F(1,35) = 9.42, *p* = 0.0041].

#### 450–550 ms

Up to this point, melody conditions were distinguishable only in virtue of the onset timing of both the music and language violation effects (earlier for unfamiliar melody conditions). In the 450–550 ms window, unfamiliar melody conditions showed only language effects [Correctness: F(1,35) = 31.13, *p* < 0.0001; Cor x AP: F(1,35) = 5.99, *p* = 0.0196; Cor x AP x Lat: F(2,70) = 3.31, *p* = 0.0453], and no Key effects or interactions.

In contrast, familiar melody conditions demonstrated both Correctness effects [F(1,35) = 16.40, *p* = 0.0003] as well as interactions of Correctness and Key [F(1,350 = 6.09, *p* = 0.0186] and Correctness x Key x AP [F(2,70) = 4.17, *p* = 0.0486]. Follow-up analyses over anterior and posterior ROIs separately showed only a main effect of Correctness over anterior ROIs [F(1,35) = 22.04, *p* < 0.0001], while posterior ROIs yielded both a Correctness effect [F(1,35) = 6.04, *p* = 0.0191] and a significant Correctness x Key interaction [F(1,35) = 11.67, *p* = 0.0016]. As can be seen in Fig. 2 (top left panel), this interaction was driven by the posterior negativity for familiar melody violations when sentences were correct and the fact that *this effect did not additively combine with the linguistic N400* – note the similar profiles of N400 responses for language-only and double violations in Fig. 2, bottom right panel). Examining this pattern within the levels of Correctness showed this negativity to be significant over posterior ROIs when sentences were Correct [F(1,35) = 6.45, *p* = 0.0157]; but when language violations occurred, the music violation response trended in the opposite direction, with the violation slightly more positive-going, but not reaching significance [F(1,35) = 2.78, *p* = 0.1046]. A further comparison across the un/familiar melody conditions when sentences were Correct showed a Familiarity x Key interaction [F(1,35) = 4.82, *p* = 0.0348], confirming the presence of this posterior negativity was unique to the familiar melody music violations.

This could suggest *either* that the music N400 response may have been completely attenuated in double-violation cases or, alternatively, that competition across the processing streams reduced both N400 responses. However, we believe the statistical analysis licenses the former interpretation (complete attenuation of the music N400 in double violations), based on the following reasoning. **First**, the music N400 demonstrates a more circumscribed posterior distribution than the language N400 effect in single violation cases, as can be seen in the voltage maps in Fig. 2, which replicates the distribution reported in Miranda & Ullman’s study. **Second**, given this fact, if it were the case that double-violations simultaneously reduced amplitude of *both* the music *and* language N400 responses to yield the observed sub-additive profile, this ought to be evident more broadly over the scalp in reduced language N400 responses over anterior ROIs as well (given its generally broader distribution).

However, this is not the pattern we see in the data, as discussed above: here we find a three way Correctness x Key x A/P interaction that corresponded to a Correctness x Key interaction *only over posterior ROIs* – anterior ROIs in contrast showed only a main effect of Correctness. This strongly suggests that the sub-additive effect was not due to a downward modulation of contributions from *both* language and music processing to the observed double violation N400 amplitude, but rather that the concurrent language violation completely attenuated the music N400 response. This is also consistent with the observed similarity of the language N400 responses in single and double violations conditions.

To summarize the important findings in this 450–550 ms latency range: the Correctness x Key interaction in this time-window reveals a sub-additive N400 response for familiar double violations whereas no such interaction manifested for unfamiliar melodies. In Fig. 3 (left and middle plots) N400 effect difference waves for music, language, and double-violations are shown for both familiar and unfamiliar melodies, plotted against the predicted additive response profile. Again, if the language and music N400 responses were independent, we would expect an additive profile: there would be no Correctness x Key interaction for familiar melodies, and the sum of the language & music difference waves (indicated by dotted black curve in Fig. 3) would not differ from the actual double-violation case, contra to fact. As argued above, the overall patterns are consistent with the interpretation that this state of affairs corresponds to a complete attenuation of the music N400 in double-violation cases.

#### 550–900 ms

Finally, in this late latency range both language and music violations yielded anterior negativities and posterior positivities as evident in the robust Key x AP [F(1,35) = 26.67, *p* < 0.0001] and Correctness x AP interactions [F(1,35) = 37.82, *p* < 0.0001] between 550–900 ms. There were no interactions between Key and Correctness involving these later effects. However, melody Familiarity did modulate the topography of the Key effects [Familiarity x Key x AP: F(1,35) = 4.13, *p* = 0.0497; Familiarity x Key x Laterality: F(2,70) = 4.07, *p* = 0.0243]. In particular, like the earlier RAN-type negativities, unfamiliar melody late anterior negativities (N500) showed no indication of lateralization, while familiar melodies were right lateralized. These lateralization differences can be seen most clearly in the double-violation ERP responses in Fig. 2, where these effects were particularly large (due to the additivity of apparently *independent* language and music late anterior negativities).

#### General summary

The results of our experiment can be summarized as follows. ***First***, both music only and language only violations replicated previously reported patterns. Violations of Key for both melody types elicited RANs followed by later N500 and P600 effects, while only familiar melodies yielded the additional posterior negativity (N400) which emerged following (and slightly overlapping with) the RAN, just as reported in M&U^24^ (though differing from M&U^24^ in onset timing, see below). The conceptual semantic violations in the visually presented linguistic stimuli also replicated previously reported patterns, including robust N400 effects followed by P600s and anterior negativities (see Steinhauer *et al*.^51^). Two findings from the original music only study did not replicate here, namely the anterior P3a and the larger P600 for familiar melody violations. The absence of these previously reported effects, as we discuss below, is relevant to our preliminary interpretation of the present data.

***Second***, in terms of cross-domain interference effects, **four** findings (I-IV) are relevant:

(I)Onset delay of the language N400. Though language N400 effects did not show amplitude modulations tied to concurrent music processing, onset timing was impacted by (un)famliarity, independent of presence/absence of music violations. The language N400 demonstrated an onset delay when the corresponding melody was familiar (Fig. 3, right panel).

(II)Onset delay of RAN/N400 complex for familiar melodies. Familiar music only violations also demonstrated an onset delay. In the previous study testing these melodies in isolation^24^, familiar and unfamiliar conditions elicited RANs with indistinguishable onset timing, with the familiar melody N400 effect following (and slightly overlapping) the RAN. Here, though the RAN and N400 effects for familiar melodies occurred together in sequential manner closely resembling the previous report, this complex of responses did not arise until 350–550 ms, whereas the RAN for *unfamiliar* melodies was already detectable in the 150–250 ms latency range.

(III)Sub-additive N400 response in familiar melody double-violation conditions. If music and language N400 responses were independent, we would expect to see an additive response profile. For familiar melodies, additivity would present with double violation N400s, expected to be larger than the language only N400 response, with no statistical interaction (i.e., just Key and Correctness main effects). However, because the unfamiliar melodies should not elicit the music N400 response, in the unfamiliar case we would expect to see similar if not identical N400 responses whether there was a concurrent music violation or not. While this is exactly what we see for unfamiliar melodies, the music N400 effect for familiar melodies appears to have been suppressed in double violations, yielding indistinguishable N400 amplitudes for familiar and unfamiliar melody condition language-only and double violation cases. In the familiar melody case, this corresponds to the crucial Key x Correctness interaction that was one of our anticipated possible findings.

(IV)Unfamiliar melody RAN effect was delayed in double violations. Presence of linguistic semantic violations suppressed/delayed the RAN for unfamiliar melodies. This was not an anticipated outcome of the present study, though as we discuss below it may not be without precedent either (e.g., see Loui *et al*.^48^).

## Discussion

The present data make a strong case that the posterior negativity for familiar melody violations documented in Miranda and Ullman (2007) is best viewed as a genuine N400 response. Both familiar and unfamiliar melody conditions here replicated music-syntactic violation responses (RAN and later N500/P600 effects), and only familiar melody violations yielded an additional N400-like negativity directly following the RAN.

Nailing down the functional significance of this component is an important finding on its own. We will henceforth refer to this effect as the **music N400** (**mN400**), as our data warrant removal of the ?-mark from M&U’s “N400?”. Concurrent auditory processing of the familiar melodies that elicit this mN400 response delayed the onset of the language N400 effect independent of the presence/absence of music violations (see Fig. 3, right panel, and scalp voltage maps for language N400 responses in Fig. 2). This N400 onset delay was seen relative to the unfamiliar melody case and in absence of any other modulations of the N400 in the unfamiliar conditions. This pattern serves to replicate previous reports that music syntactic violations ***on their own*** do not influence language N400 effects^12,20^. We take this dissociation to indicate competition for resources in the familiar melody conditions only, involving the management of the activation of language and music LTM semantic memory systems.

Importantly, the language N400 onset delay for familiar melody conditions is not the only relevant finding in these data. When sentences were well-formed, the RAN/mN400 complex for violations in familiar melodies was delayed. In Miranda and Ullman (2007), familiar and unfamiliar melody RAN effects showed equivalent onsets (~150–270 ms) with the familiar melody mN400 onset only slightly later (220–380 ms). Here, the familiar melody RAN did not emerge until 350–450 ms with the mN400 arising at 450–550 ms. That is, familiar melody violation responses were delayed by concurrent language processing in the present study by as much as ~200 ms (judging by comparison to the previously reported data). It may be no accident that the magnitude of this delay in familiar melody violation responses roughly corresponds with estimates of the timing of early aspects of lexical access^55,56^.

Finally, the fact that our familiar melody double-violations yielded a sub-additive N400 response is also strongly consistent with M&U’s interpretation of their posterior negativity as a *bona fide* N400 effect. Further, as noted above (see 450–550 ms time-window report in Results), the pattern of effects suggests that this sub-additive response is best understood as a complete suppression of the music N400 response in double-violation cases. As can be seen in the data visualizations (Fig. 2), and as our statistical analysis showed, the language N400 response appears completely unperturbed by the concurrent music violation (note the similarity of average ERP curves for language only (blue traces) and double violation cases (green traces) across the scalp for familiar melodies in Fig. 2, and the overall similarity to the corresponding effects for the unfamiliar melodies).

Before we turn to other effects we have documented here, and possible ways to interpret these patterns overall, it is important to note that the present findings concerning the N400 and language/music processing differ in important ways from previous findings concerning language/music syntactic processing and the corresponding LAN/RAN response profiles. Whereas previous work^12^ showed a LAN (language effect) attenuation in double violation cases, here we show different kinds of cross-domain influences in both directions (e.g., familiarity of melodies delayed language N400 onset; double violations attenuated music N400 amplitude; concurrent language processing delayed the RAN/mN400 onset, etc.).

In addition, another important pattern documented here involving an influence of concurrent language processing on music is the early sub-additive response over anterior scalp regions for the *unfamiliar* melody double-violations, which appears to be best described as indicating an early suppression/delay of the RAN. This seems important to consider relative to the fact, just mentioned above, that *both* the RAN and mN400 effects were delayed in the familiar melody condition when sentences were correct (compared to M&U’s music-only results), and that the RAN was generally later in onset for the familiar cases (see scalp voltage maps in Fig. 2). The only early onset RAN effect we find here was for single (music) violations when melodies were unfamiliar. How can we interpret these patterns?

Since the language violations we tested here turned on lexical/conceptual semantic properties of target words, the fact that these violations would influence the RAN effect for unfamiliar melodies could be seen as an unexpected result based on previous findings^12,20^. Similar considerations arise for the delay of the RAN for familiar melody violations that arose independent of sentence correctness. Simultaneous demands on LTM access/retrieval across processing streams could be appealed to in order to explain the delay of the mN400 (and the language N400), but why should the RAN effects also be delayed? And why should this RAN delay occur *uniformly* for familiar melodies with no apparent influence of language Correctness, but show an interaction profile for the unfamiliar conditions with RAN delay only when both streams were simultaneously violated?

Given these patterns, one might be tempted to turn to accounts that appeal to demands on general attention^10^. However, such an explanation would not easily account for the full range of findings in the present study (in addition to other worries that can be raised about such accounts, see Slevc & Okada^7^ and Kunert *et al*.^57^). It is possible that for unfamiliar melodies, when sentences were well-formed, there were only weak demands on attentional resources and so the music RAN effect showed an onset timing consistent with the previous music-only study. In contrast, when semantic violations were present, this could have increased the processing load, attenuating the task irrelevant auditory channel, thus suppressing/delaying the RAN. It has already been demonstrated elsewhere^48^ that RAN effects can be suppressed when attention is directed away from music, so this part is plausible.

But we might then ask if this reasoning can explain why the RAN effect was delayed for *familiar* melodies when there was *no* concurrent language violation. It is possible that familiar melodies generally capture less attentional resources in virtue of their *predictability*. It is known, for example, that attention and predictability can have opposite effects on early evoked responses (e.g., smaller N1 effects for predicted items, larger N1 effects for attended items^58^). The predictability of familiar melodies may thus cause less attention to be given to the auditory stream, mirroring the previous argument of attenuation of the auditory channel by semantic violations in unfamiliar melodies (and creating a similar delay in the RAN effect). But if familiar melodies were less well-attended in virtue of their predictability, why would their concurrent processing cause a delay in the language N400 effects? This latter aspect of the response pattern suggests that attention was directed *away* from the visual/language processing stream by the presence of familiar melodies (i.e., that they demanded *more*, not *less*, attention).

Likewise, several other aspects of our ERP data, relative to patterns in Miranda and Ullman (2007), seem to weigh against any *simple* appeal to attention here. First, that our task directed attention away from the music stimuli is consistent with the absence of the P3a effect for familiar melodies found in the previous music-only study *and* with the fact that familiar and unfamiliar melodies elicited equivalent P600 effects (whereas previous findings showed larger P600s for familiar melody violations^24,39^). Reduction of P600s as a function of presence/absence of task-relevant attention is expected based on other prior work (see review^49^). Thus, asymmetries in the response profiles, plausibly linked to attention differences across familiar and unfamiliar melodies in the music-only study^24^, could be said to have been neutralized here where the task directed attention to the visual/language processing stream.

This neutralization of likely markers of attentional asymmetries across melody types may also have very early correlates in the ERP record. An undiscussed pattern in M&U’s data (Fig 3 & 4 in M&U^24^), pertains to the early auditory P1-N1-P2 complex, where P2 responses were noticeably larger for unfamiliar compared to familiar melodies. In fact, inspection of their data suggests amplitude differences as large as ~2 μVs related to (un)familiarity, though these apparent differences were not analyzed. This may be thought of in terms of the attention/prediction trade-off mentioned above, with the predictability of familiar melody continuations resulting in lower demands on early perceptual level processing.

Here, in contrast, auditory and visual N1-P2 effects superimposed, yielding larger amplitudes in general but with no indication of larger P2 amplitudes when melodies were unfamiliar (Fig. 2). Like the absence of the P3a and the familiar/unfamiliar P600 amplitude differences, these observations are consistent with the absence of general attentional asymmetries between familiar and unfamiliar melodies in the current study that were otherwise present when the melodies were processed in isolation and attended to in a task-relevant way.

What must be accounted for here is the *symmetry* of interference for familiar melodies, and the *asymmetry* of the interference when melodies were unfamiliar. It may be that an account based in considerations involving attention can be reconciled with views positing shared syntactic processing resources within models that view working memory as attention-regulated activation of long term memory (LTM) representations (see LaRocque *et al*.^59^ for overview).

In the unfamiliar melody cases, since detection of the music violations does not implicate activation of specific long term memory representation (i.e., of the “musical lexicon”, in the sense similar to Peretz *et al*.^60^), there is no conflict with the sub-systems at work in language processing involving prediction and access/retrieval of lexical/conceptual semantic information associated with incoming words when sentences were well-formed. Accordingly, RAN effects for unfamiliar melodies are registered with a timing similar to when music is processed in isolation, so long as there are no disruptions in the language processing stream. In contrast, for familiar melodies, attentional control mechanisms of LTM activation must necessarily already be subjected to simultaneous demands across the language and music streams even prior to encounters with target notes/words in our paradigm.

Development of this line of explanation seems capable of accounting for the bi-directional effects on the N400 for language and music in the familiar melody conditions. If correct, this would also suggest that the RAN for familiar melodies is not automatically triggered by the deviant out-of-key notes independently of the processes involved in access/retrieval of corresponding musical LTM representations, at least in circumstances when music is not attended to. From this perspective it would be important to determine what effect a task directing attention to the auditory/music stream would have. In principle, this could result in bringing the onset latency of familiar and unfamiliar melody RAN effects back into their previously reported alignment. An open question would be whether this would also result in a similarly earlier onset for the mN400, more consistent with the timing reported in the previous music-only study, or not (we are investigating these questions directly in the context of our larger scale study).

If RAN effects are generally sensitive to attention in this way (consistent with Loui *et al*.^48^), then the suppression/delay of the unfamiliar melody RAN when there was a concurrent language violation could be given the simple treatment sketched above. Given the lack of competition between the streams in terms of attentional control of the activation of LTM representations, presence of a language violation captures attentional resources unhindered by demands from the music processing stream, resulting in the delay of the RAN in double-violations, but no influences in the other direction (i.e., no effects of unfamiliar melody processing on language). In the familiar melody double-violation conditions we expect a different pattern, as both streams involve simultaneous disruptions of activity underlying the N400. In this case, having attentional resources swamped by processing of the language violation could be expected to suppress the mN400 response – precisely the pattern evident in our data.

Of course, this line of interpretation requires further fleshing out, and several follow-up inquiries are required to demonstrate how robust (replicable) and how general these N400 interference effects may be (see below). Our point here is simply that an approach that takes attention into account in formulating the nature of cross-domain effects concerning working/long-term memory and language/music relationships seems to be recommended. That is, even if we concur with arguments that *general* appeals to attention are *insufficient* on their own to provide an account of cross-domain interference effects^7,57^, it may be that appeals to attention are nonetheless *necessary* for any adequate account of such patterns.

In addition to the open questions discussed above, as mentioned in §1 many but not all of the familiar melodies we adopted from the M&U study have associated lyrics. Thus, it will be important to determine whether the presence of the mN400 (and/or the cross-domain interaction patterns) depend on this feature or not. Previous work has provided documentation of both dissociations between melody and lyric processing^54,60^ as well as their tight linkage^61^. However, it is important to point out that this issue does not diminish the interest and importance of the present findings. Indeed, showing as we have here for the first time that N400s can indeed be elicited in connection with isolated music stimuli, and can in addition yield interference profiles in paradigms like the one employed here, opens up a range of possibilities regarding future follow-up work on the nature of musical LTM, which is of course multi-dimensional (linking to semantic, episodic, and emotional/affective memory systems, in addition to the systems supporting LTM of lyrics). This finding should be significant also for studies of musical memory in special populations (e.g., in patient groups, examinations of age-related cognitive changes, etc.^62–66^). Further, additional questions arise about *how general* this type of cross-domain N400 interaction may be^67^, and what impact the potential generality of such interactions would have on our understanding of musical semantics^42,68^. For example, N400-like effects have also been reported for familiar versus unfamiliar environmental sounds^69^ and it is an open question as to whether such effects would cause interference with language (or music) N400 responses as well (see also work examining N400 responses to words versus environmental noises^70,71^).

Finally, the fact that it is possible to elicit such N400 interference profiles across these cognitive domains (and modalities) suggests possible opportunities for leveraging these findings in efforts to adjudicate between ongoing disputes concerning the etiology of this ERP response (access/retrieval? binding? integration? inhibition?), and how it relates to general concerns about working memory and attention (see also discussion in Kutas & Federmeier^26^ regarding the N400 and attention). Our findings also recommend connecting this type of inquiry with work on unimodal versus cross-modal attention^72^ and multi-sensory integration more broadly^73^.

## Methods

### Participants

Data reported reflects *N* = 36 (20 M; 16 F; age 18–31; *M* = 21.08, *SD* = 2.77). All were right handed (assessed by modified version of the Edinburgh Handedness Inventory^74^), had normal or corrected to normal vision, normal hearing, and no history of neurological pathology. Music experience ranged from 0–30 years singing or playing an instrument (*M* = 7.76 years; assessed by questionnaire adapted from Wei Looi^75^). Participants’ scores ranged from 1.00 to 4.86 on the Innovative Musical Aptitude subscale of the Brief Music Experience Questionnaire^76^. This subscale measures musical performance ability (1–5 low-to-high experience scale). Fourteen participants (~39%) reported they currently play an instrument or sing. All participants gave informed written consent and were given research credit or were paid for participation.

### Materials

#### Musical stimuli

These were drawn from M&U^24^. *Familiar* melodies were chosen from six different categories: traditional/folk, children’s music, patriotic songs, holiday songs, classical music, and pop/theme music. Many, although not all, of these melodies have lyrics associated with them. *Unfamiliar* (novel) melodies were composed to be pair-wised matched to familiar melodies. Of the 120 melodies in M&U^24^, we used 112 (8 excluded due to very fast tempo). Individual note onsets were extracted for use in timing the visual word-by-word sentence presentation. Melodies were grouped together in sets of four, creating twenty-eight groups of melodies (groups had similar total number of notes).

#### Sentence stimuli

These were constructed around object relative clauses containing target VERBS, created in 4-tuples (e.g., the ball that the boy has KICKED/*BAKED; the pie that the boy has *KICKED/BAKED) to rotate target words across conditions. 4-tuples were duplicated and edited to allow for both inflected and uninflected target verbs (e.g., …the ball that the boy will KICK…), creating a total of 56 8-tuple groups of sentence frames.

#### Melody-sentence pairing and list creation

The melody list was duplicated so that each of the 28 groups of melodies contained two sets of its four unique melodies. Each group of melodies was randomly assigned to two 8-tuples of object-relative clause frames, such that each melody was assigned to two matched pairs of correct/violation sentence frames (one from each 8-tuple). Each sentence frame pair was then matched with each of the four versions of that melody. This created 16 unique items for each melody, for a total of 64 unique items for each group of melodies. This was done for all 28 groups of melodies, yielding 1792 unique items (sentence-frame/melody pairings).

Sentence beginnings/endings were added to create a context for each relative clause frame. Critical target verbs were aligned to target notes of the melodies; each syllable of text occurred with one melody note. Target verbs and the immediately preceding word were always monosyllabic.

The master list of sentence/melody pairings was factored into eight presentation lists, each consisting of 224 sentence-melody pairs (28 per condition in the 2 × 2 × 2 (Familiarity x Correctness x Key design). Items were distributed across lists such that each list contained both a familiar and unfamiliar version of each melody with its matched sentence. In-key and Out-of-key versions of any given melody never appeared in the same list. Unfamiliar versions of each melody always preceded the familiar one. Items were rotated such that no list contained both the correct/violation version of any sentence and were pseudo-randomized.

### Procedure

All procedures in the present study were carried out in accordance with relevant guidelines, precisely as specified in the experimental protocol approved by Stony Brook University IRB.

#### Session 1: EEG data collection

Participants were seated in a booth facing a computer monitor while wearing ear-insert earphones. Sentence-melody pairs were presented with words appearing one at a time in the center of the computer monitor. Each word was presented at note onset with timing determined by the length of the note, until it was replaced by the next word (at onset of following note). Both target and pre-target notes always had a uniform duration of 600 ms. Participants were made aware that they may hear some familiar melodies along with some unfamiliar ones, and that some melodies may include “out of key” or “sour” notes. Participants were instructed, however, to evaluate only the visual/linguistic stimuli, producing an end of sentence judgment for all trials. Sentences were judged as “good” or “bad”, indicated by a mouse click.

#### Session 2: “Name That Tune”

Participants returned for a second session (always within less than one week from session 1) during which Familiar Correct and Unfamiliar Correct versions of the musical stimuli presented in Session 1 were re-presented, but without sentence stimuli. Melodies were played over speakers and presented in the same order as in Session 1. Participants indicated by mouse click songs they recognized and were asked to say the name of the tune out loud if identified. If they did not know/remember the name of the song, but were able to give other information (lyrics, semantic category, etc), they were instructed to provide this. Familiarity judgments were recorded automatically, while any comments supplied by the participant were recorded manually by the experimenter.

### EEG recording, data processing, and analysis

Scalp EEG was recorded from 32 electrodes plus H/VEOG (512 Hz sampling rate), referenced offline to averaged left/right mastoids (BioSemi Active2 amplifiers; online bandpass of 0.01–125 Hz). Trials contaminated with eyeblinks or other artifacts were excluded automatically. All channels were filtered offline (0.1–30 Hz) and single subject waveforms were averaged over 1200 ms epochs following the target note, including −200 to 0 ms baseline correction interval.

Repeated measures ANOVAs were conducted to analyze ERP data over midline and lateral regions of interest (ROIs) in a 2 × 3 grid with two levels of Anterior/Posterior (AP) and three levels of Laterality (left/mid/right) as follows: Left-Anterior = AF3/F3/F7, Mid-Anterior = FP1/FP2/Fz, Right-Anterior = AF4/F4/F8, Left-Posterior = CP5/P3/PO3, Mid-Posterior = Cz/CP1/CP2/Pz/O1/Oz/O2, Right-Posterior = CP6/P4/PO4. All analyses of mean amplitude measures within given time-windows (150–250, 250–350, 350–450, 450–550, 550–900 ms) were carried out over these ROIs. Analyses included the language factor Correctness (Correct/Violation) and the music factor Key (In-key/Out-of-key). These analyses were first carried out for familiar and unfamiliar melodies separately, to probe for predicted effects. Additional analyses including the factor Familiarity were carried out to demonstrate statistical interactions where relevant, in particular concerning the presence/absence of the music N400 response. Greenhouse-Geiser corrections were performed where relevant (corrected *p*-values are reported throughout). Onset timing of language N400 effects were examined, including peak amplitude and both peak latency and (50%) fractional peak latency measures, analyzed using a jackknife method^77^. Reported F-values for the jackknife analyses were adjusted by dividing by (N-1)^2^. Sequence final acceptance rates (% accepted) for judgment task and familiarity judgments (% judged as familiar) in the follow-up *Name That Tune* task, were also examined with repeated measures ANOVA.

### Ethics

All participants provided informed written consent. The study was approved by the Stony Brook University IRB.

## Data availability

Datasets supporting this report are included as electronic supplementary information (see SI-Overview).
